# Time trends in single versus concomitant neck and back pain in finnish adolescents: results from national cross-sectional surveys from 1991 to 2011

**DOI:** 10.1186/1471-2474-15-296

**Published:** 2014-09-05

**Authors:** Minna Kristiina Ståhl, Ashraf Abdel Salam El-Metwally, Arja Hannele Rimpelä

**Affiliations:** Department of Physical and Rehabilitation Medicine, Hatanpää Hospital, Hatanpäänkatu 24, PO Box 437, Tampere, 33101 Finland; University of Helsinki, Hjelt Institute, PO Box 40, Helsinki, 00014 Finland; Department of Epidemiology and Biostatistics, College of Public Health, King Saud Bin Abdulaziz University for Health Sciences, PO Box 22490, Riyadh, 11426 Saudi Arabia; Aberdeen Pain Research Collaboration (Epidemiology Group), Institute of Applied Health Sciences, University of Aberdeen, Aberdeen, AB25 2ZD UK; School of Health Sciences, University of Tampere, Tampere, 33014 Finland; Department of Adolescent Psychiatry, Tampere University Hospital, Pitkäniemi Hospital, Pitkäniemi, 33380 Finland

**Keywords:** Neck pain, Low back pain, Concomitant neck and low back pain, Spinal pain, Musculoskeletal pain, Adolescent, Time trend, Serial cross-sectional design, Prevalence

## Abstract

**Background:**

Previous studies, in late 20th century, suggest an increase in the prevalence of neck pain and low back pain among children and adolescents, when neck and low back pain were studied separately. This study investigated time trends in adolescent spinal pain between 1991 and 2011 by classifying pain into the following three classes: neck pain alone, low back pain alone, and concomitant neck and low back pain.

**Methods:**

Representative samples of 12 to 18-year-old Finns were sent a questionnaire in 1991, 1999, 2001, 2003, 2005, 2007, 2009 and 2011. Information was gathered about the frequency of neck and low back pain with a six-month recall period. Statistical methods used included descriptive analysis, and generalized linear models.

**Results:**

The total number of respondents in these eight comparable cross-sectional surveys was 51 044 with a response proportion of 64%. The prevalence of concomitant neck and low back pain showed a steady increase from 1991 to 2009/2011; the prevalence almost quadrupled among 12-14-year-olds girls (from 2% to 7.5%), and more than doubled among 12-14-year-old boys (from 1.6% to 3.8%), and among 16-18-year old boys (from 4.2 to 9.9%) and girls (6.9% to 15.9%). The prevalence of neck pain alone only increased in the 1990s (e.g. among 16-18-year-old girls 22.9% in 1991, 29.2% in 1999, and 29.5% in 2011), while the prevalence of low back pain alone remained relatively constant during the last two decades (e.g. among 16-18-year-old girls 4% in 1991, 3.1% in 1999, and 3.7% in 2011).

**Conclusions:**

Concomitant neck and low back pain has constantly increased in the last two decades among adolescents, while single neck pain has only increased in the 1990s. Single low back pain has remained relatively constant. Thus, earlier detected increase in low back pain in the 1990s was explained by the increase in concomitant neck and low back pain. Differences in the time trends in the three pain conditions might suggest, at least partly, different risk factors and aetiology for single- and multisite spinal pain among adolescents. This hypothesis needs further investigations.

**Electronic supplementary material:**

The online version of this article (doi:10.1186/1471-2474-15-296) contains supplementary material, which is available to authorized users.

## Background

The prevalence of neck pain and, to a lesser extent, low back pain among children and adolescents increased in the late 20th century [1]. Given the postulated link between neck and back pain symptoms in childhood, adolescence, and adulthood, this might pose a future health challenge [2–5]. Whether this observed trend has continued in the new millennium is unknown.

The past two decades have witnessed changes in environmental exposures that could have influenced occurrence of neck and low back pain. Such potential changes among adolescents included unfavourable shifts in leisure-time activity patterns; decrease in physical activity and tremendous increase in the use of information and communication technology [6]. Such behavioural changes have been suggested to be contributing factors to the increased occurrence of neck and low back pain, mainly through an assumed increase in repetitive movements and static postures causing localised muscle fatigue and pain [7]. Furthermore, a secular increase has been noted in psychosomatic and stress symptoms, and sleep disturbances [8–10]. These changes might suggest a continuing increase in neck and low back pain in adolescents.

In earlier studies, time trends of neck and low back pain have been studied separately whereas changes in concomitant neck and low pain have not been studied. Neurobiological processing of negative emotions and pain resemble each other [11], and it has been suggested that frequent pain symptoms in childhood and adolescence should be considered a potential general pain disorder reflecting psychological stress, rather than merely a localised body disorder [12, 13]. On the other hand, the Finnish twin studies among early adolescents showed that genetic factors play the most important role in the susceptibility to neck pain [14], whereas low back pain and widespread pain are related to common and unique environmental factors in early adolescence [15, 16]. Based on this observation and taking into account changes in the risk factors it is possible that time trends in concomitant and single neck and low back pain differ from each other.

Altogether, only few studies have investigated adolescents’ neck pain with other musculoskeletal pain. In a 4-year follow-up study of originally pain-free preadolescents, neck pain occurred more often with other musculoskeletal pain, most often with lower limb or axial pain, than as a single pain. [17]. In a cross-sectional study among 14-year-old Australians, single neck and low back pain were overall more common than concomitant neck and low back pain [18]. It is not clear, whether single and concomitant neck and low back pain are separate entities, overlap or occur consecutively in adolescents.

This study investigated time trends from 1991 to 2011 in three types of spinal pain: neck pain alone, low back pain alone, and concomitant neck and low back pain among 12 to 18-year-old Finns.

## Methods

### Subjects

The Adolescent Health and Lifestyle Survey is an on-going (since 1977) nationwide monitoring system on adolescent health and lifestyle in Finland. Data is collected every second year from 12 to 18-year-old Finns. Here we used data from 1991, 1999, 2001, 2003, 2005, 2007, 2009, and 2011, because only in all those surveys neck and low back pain were assessed with separate questions. The sampling strategy, and data collection methods were similar across the survey years. Samples of 12, 14, 16, and 18-year-old Finns (mean age 12.6, 14.6, 16.6, and 18.6 years) born on certain dates in July (in some age groups, dates from June and August were used to avoid using the same persons in different years) were drawn every survey year from the National Population Registry Centre. A questionnaire, that included multiple questions related to health and health behaviours, was sent in February with a return envelope and pre-paid postage. In 2009 and 2011, the option of completing the questionnaire through the Internet, using a personal username and password, was provided. Two additional attempts were made to contact non-respondents.

### Measurements

Each survey year the questionnaire had approximately a hundred questions. The surveyed areas were health and health complaints, use of alcohol and tobacco products, physical activity, and sleep behaviour as well as socio-economic background, family and school performance. The core areas were kept the same over the survey years while part of the questionnaire varied including e.g. violence, meal patterns, gambling or hopes and worries for the future. Each study year, the new questions added to the questionnaire were pretested in a sample of schoolchildren.

Each survey year the questionnaire included the following two questions about spinal pain: “Have you had low back pain during the past 6 months?” and “ Have you had neck pain during the past 6 months?” In the latter question, the Finnish language refers to the anatomical area including the neck spine, occiput, and other structures covered by the upper trapezius muscles. Answer options for both questions were as follows: seldom or not at all, about once a month, about once a week, and almost daily. Both variables were dichotomised; pain frequency categories ‘seldom or not at all’ and ‘about once a month’ were merged and rephrased as ‘no or infrequent pain’, and ‘about once a week’ and ‘daily pain’ were also merged and rephrased as ‘at least weekly pain’. The latter two phrases were merged mainly because of the very small number of respondents with daily symptoms. For analyses, four variables were created: 1. No or occasional neck and low back pain 2. Neck pain alone at least weekly, 3. Low back pain alone at least weekly, and 4. Concomitant neck and low back pain at least weekly. Test-retest reliability of the questionnaire for detecting those reporting pain at least weekly was evaluated earlier in a subsample of 14-to 16-year-old adolescents from the 2003 cohort with a kappa coefficient of 0.56 for neck pain, and a similar coefficient of 0.56 for low back pain [7]. Other tests of validation were not performed. Our study follows the guidelines of STROBE (see Additional file 1).

### Analysis of non-respondents

The possible effect of a decrease in response proportions on the prevalence of pain symptoms was investigated indirectly, since information from non-respondents was not available. The respondents were categorised into three groups based on how promptly they returned the questionnaire (original, first re-inquiry, and second re-inquiry). It was assumed that the later the person answered, the more he/she resembled a non-respondent. We used the years 1999, 2003, and 2011. Differences between the three respondent groups were small and not systematic over the years or over the age or gender groups.

### Ethics

This study follows the ethical principles of the Declaration of Helsinki; subjects were informed of the aims, methods, voluntary participation, privacy, and confidentiality of the collected information. The study protocol was approved by the The Ethics Committee of Tampere Region (reference “Lausunto 2/2010).

### Statistical methods

Descriptive values are shown in prevalence proportions (%) with 95% confidence intervals. Generalized linear models with appropriate distribution and link function was used to check the statistical significance of the linearity of trends in the three pain groups. The time difference between the surveys years was taken into account. Relative rates were estimated by using generalized linear models with binomial family and log link. The prevalence of neck pain alone, low back pain alone and concomitant neck and low back pain in 1991 was used as a reference in calculating the relative risks for the pains in 1999, and in 2011.

## Results

Table 1 shows the number of respondents for each survey year by age and gender. Response proportions ranged between 75% in 1991 to 46% in 2011 with a mean percentage of 64% for all assessments combined. Response proportions were generally higher for girls than for boys regardless of age.

**Table 1:** **Number of respondents (N) and response proportion (%) by survey year, gender and age**

						Year				
		1991	1999	2001	2003	2005	2007	2009	2011	Total
Boys										
12-14 y	N	1559	1583	1533	1447	1420	1136	1010	893	10581
	%	72	73	65	66	62	54	48	43	61
16-18 y	N	1867	2194	1613	1561	1443	1205	1205	932	12020
	%	64	64	55	55	51	43	41	31	51
Girls										
12-14 y	N	1691	1685	1831	1626	1560	1402	1313	1073	12527
	%	83	83	77	77	73	70	66	57	73
16-18 y	N	2354	2608	2069	2085	1896	1874	1838	1538	16262
	%	83	81	77	76	71	70	64	54	72
Total		7471	8070	7046	6719	6319	5617	5516	4566	51044
		75	75	68	68	64	59	56	46	64

**Figure 1:**
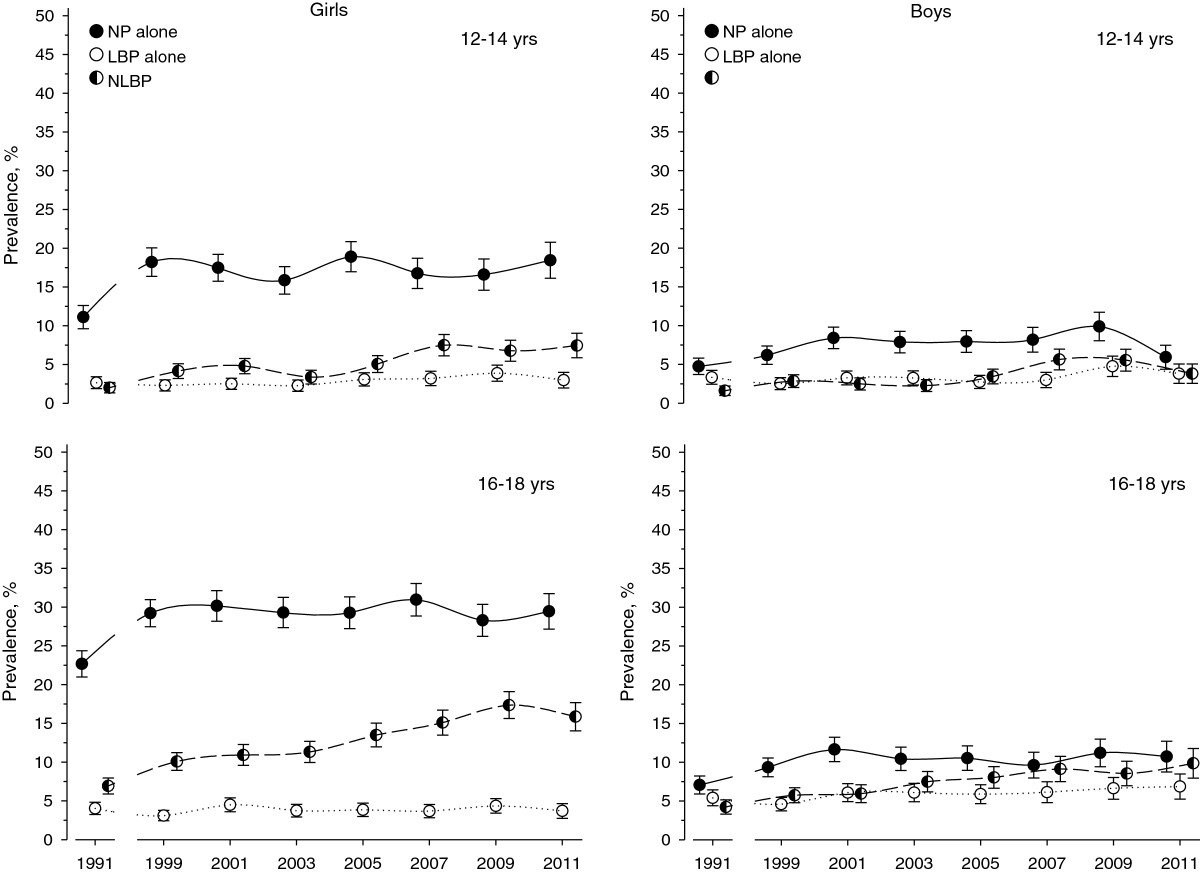
**Time trends in three different types of spinal pain between 1991 and 2011; neck pain (NP) alone, low back pain (LBP) alone, and concomitant NP and LBP (NLBP) in 12-14- and 16-18-year-old Finnish girls and boys.**

Table 2 and Figure 1 illustrate the changes in the prevalence of neck pain alone, low back pain alone, and concomitant neck and low back pain from 1991 to 2011 by age and gender. All three types of pain were more common among girls than boys, and among older adolescents compared to younger ones in all eight surveys. Neck pain alone was the most common in all age and gender groups in all surveys. Statistically significant linearity was observed in all trends in neck pain alone, and concomitant neck and low back pain, but only in half of the trends in low back pain alone (Table 2). However, a closer observation (Tables 2 and 3, and Figure 1) shows that the prevalence of neck pain alone increased from 1991 to 1999 in girls and to 2001 in boys, after which the trend levelled off. The increasing trend in concomitant neck and low back pain continued through the whole study period to 2009/2011 in all age and gender groups. Table 3 shows the relative risks (RR) for different pains in 1999 and in 2011 compared to the reference year 1991. From 1991 to 2011 the prevalence of concomitant neck and low back pain almost quadrupled among 12 to 14-year-old girls (RR 3.7), and more than doubled among other groups (RR 2.4 among 12-14-year-old boys, and RR 2.3 among 16-18-year-old boys and girls). The prevalence of low back pain alone remained fairly unchanged over the study period in all age and gender groups.

**Table 2:** **The prevalence (%) of neck pain alone (NP), low back pain alone (LBP), and concomitant neck and low back pain (NLBP) by survey year, sex, and age**

Survey year									*P for linear
	1991	1999	2001	2003	2005	2007	2009	2011	trend
Boys									
12-14									
NP alone	4.8	6.2	8.4	7.9	8.0	8.2	9.9	5.9	< 0.001
LBP alone	3.3	2.5	3.3	3.3	2.8	3.0	4.8	3.8	0.11
NLBP	1.6	2.8	2.5	2.3	3.5	5.6	5.5	3.8	< 0.001
16-18									
NP alone	7.1	9.3	11.7	10.4	10.5	9.6	11.2	10.7	< 0.001
LBP alone	5.4	4.6	6.1	6.1	5.9	6.1	6.6	6.9	0.008
NLBP	4.2	5.7	6.0	7.5	8.0	9.1	8.6	9.9	< 0.001
Girls									
12-14									
NP alone	11.1	18.2	17.5	15.9	18.9	16.8	16.6	18.5	< 0.001
LBP alone	2.7	2.3	2.5	2.3	3.1	3.2	3.9	3.0	0.023
NLBP	2.0	4.2	4.8	3.4	5.1	7.5	6.8	7.5	< 0.001
16-18									
NP alone	22.7	29.2	30.2	29.3	29.3	30.9	28.3	29.5	< 0.000
LBP alone	4.0	3.1	4.5	3.7	3.9	3.7	4.4	3.7	0.060
NLBP	6.9	10.1	10.9	11.3	13.5	15.1	17.4	15.9	< 0.000

Level of significance for linear trends is shown with P-values.

**Table 3:** **Relative risks (RR) for neck pain alone (NP), low back pain alone (LBP), and concomitant neck and low back pain (NLBP) in 1999, and in 2011 compared to the reference year 1991**

	RR (95% CI) for pain in 1999 compared to 1991 (RR 1.00)	RR (95% CI) for pain in 2011 compared to 1991 (RR 1.00)
NP alone		
Boys		
12-14	1.30 (0.97 to 1.75)	1.25 (0.89 to 1.76)
16-18	1.32 (1.07 to 1.63)	1.52 (1.18 to 1.94)
Girls		
12-14	1.64 (1.38 to 1.94)	1.66 (1.38 to 2.00)
16-18	1.29 (1.17 to 1.42)	1.30 (1.17 to 1.45)
LBP alone		
Boys		
12-14	0.76 (0.50 to 1.14)	1.14 (0.75 to 1.75)
16-18	0.85 (0.65 to 1.11)	1.27 (0.94 to 1.72)
Girls		
12-14	0.87 (0.57 to 1.33)	1.12 (0.72 to 1.75)
16-18	0.77 (0.58 to 1.03)	0.92 (0.67 to 1.27)
NLBP		
Boys		
12-14	1.77 (1.09 to 2.87)	2.37 (1.42 to 3.95)
16-18	1.36 (1.03 to 1.79)	2.33 (1.75 to 3.12)
Girls		
12-14	2.07 (1.38 to 3.10)	3.71 (2.50 to 5.50)
16-18	1.46 (1.21 to 1.76)	2.29 (1.90 to 2.76)

Calculations are based on the prevalences presented in Table 2.

## Discussion

The findings of the present study show a steady increase in the prevalence of concomitant neck and low back pain among adolescents over the last 20 years. Prevalence of neck pain alone increased only in the 1990s, whereas the prevalence of low back pain alone has remained quite stable over the past two decades.

The strengths of the present study are its fully comparable eight cross-sectional surveys over the last two decades allowing an exploration of time trends in a large and representative sample of adolescents. The symptoms, across surveys, were asked at the same time of the year; thus, the fluctuation of symptoms due to seasonal variation was excluded [19]. In addition, the frequency of neck and low back pain during the past six months was assessed, so the results refer to frequent and persistent spinal morbidities (i.e., excluding occasional pain such as menstruation pain). Since neck and low back pain were asked about only with one combined question in 1993, 1995 and 1997, data from these years could not be used in this study. The constant decrease in response proportions over the study years, especially among boys, is another limitation that might have had an influence on the reliability of the prevalence figures and estimated odds ratios. Information about the non-respondents would have been needed in order to exclude a potential selection bias. However, we did not have any information about the non-respondents, but instead attempted to indirectly investigate its potential effect by comparing the three different groups of respondents on how promptly they had returned the questionnaire (original, first re-inquiry, and second re-inquiry) with an assumption that the later the person answered, the more he/she resembled a non-respondent. Although the validity of this assumption can be discussed, our findings did not give any support for a potential selection bias, with non-significant differences in pain prevalence between early and late respondents. Several other methodological issues might have affected the internal validity of the study. The test-retest reliability of the pain questions over three weeks was only moderate [20]. In addition, the six-month recall period was quite long taking into account the fluctuating natural course of at least neck pain [5], which may have led to difficulties in frequency classification of the symptoms. Since there was no pain mannequin alongside the pain questions, the neck pain alone group might contain those who have only upper back pain, and those with concomitant neck and upper back pain.

To our knowledge, this is the first study presenting time trends for single versus concomitant neck and low back pain in adolescents. The increasing trend in low back pain in the late 20th century reported by Hakala et al. [1] was thus explained by the increase in concomitant neck and low back pain. The prevalence of all three types of spinal pain increased with age and was more common in girls than in boys, consistent with previous reports [5, 21–23]. A systemic overview of the research literature on epidemiology of adolescent spinal pain concluded that the comparison of the studies is difficult due to great variety of different pain definitions and outcome measurements [21]. We did not find any other studies that used the same pain definitions and recall period used in our study. The only other study that has investigated the prevalence of single versus concomitant neck and low back pain without asking other musculoskeletal pain was conducted by Rees et al. [17]. They investigated the 1-month period prevalence of single and concomitant neck and low back pain among 1580 (98%) 14-year-old Australians in 2000s. The prevalence of neck pain only was 17%, back pain only 13%, and concomitant neck and back pain was 18% among girls. Corresponding proportions among boys were 14%, 17% and 9%.

Our finding of an increased occurrence of concomitant neck and low back pain and a more constant occurrence of neck pain alone or low back pain alone during the last two decades could be explained by at least partly different underlying aetiologies for the two types of pain. First, the increase in concomitant pain could reflect the changes in the contemporary adolescents’ life, such as an increase in computer use [6], sleep difficulties [10], and a decrease in psychosocial wellbeing [8, 9], all of which have been found risk factors for low back and neck pain [3, 5, 7, 24–28]. The results of Rees et al. [18] support this hypothesis; concomitant neck and low back pain was associated significantly more with mental health problems than single neck and single low back pain. Second, the prevalence of neck pain alone or low back pain alone were fairly constant over the last 20 years, which could be linked to familial factors, i.e., genetic and common environmental factors, which remain more stable over time [14, 15]. Future studies aimed at investigating whether these two types of spinal pain are truly separate entities or whether they overlap or occur consecutively are needed. Furthermore, future studies should be aimed at understanding the factors that underlie the increase in concomitant neck and back pain, rather than factors that only underlie either neck or back pain.

## Conclusion

The prevalence of concomitant neck and low back pain has steadily increased over the past 20 years, while the prevalence of neck pain alone has increased only in the 1990s. The prevalence of low back pain alone has remained quite stable. Our findings suggest that the underlying aetiology for single and concomitant neck and low back pain in adolescents might, at least partly, differ. Future studies should be aimed at investigating this hypothesis more closely, and especially in relation to already identified risk factors for adolescent neck and low back pain.

## Electronic supplementary material

Additional file 1:**STROBE Statement—Checklist of items that should be included in reports of** ***cross-sectional studies (X= done, N/A = not applicable).***(DOC 79 KB)

Below are the links to the authors’ original submitted files for images.

Authors’ original file for figure 1

## References

[1] Hakala P, Rimpelä A, Salminen JJ, Virtanen SM, Rimpela M (2002). Back, neck, and shoulder pain in Finnish adolescents: national cross sectional surveys. BMJ.

[2] Brattberg G (2004). Do pain problems in young school children persist into early adulthood? A 13-year follow-up. Eur J Pain.

[3] Siivola SM, Levoska S, Latvala K, Hoskio E, Vanharanta H, Keinanen-Kiukaanniemi S (2004). Predictive factors for neck and shoulder pain: a longitudinal study in young adults. Spine.

[4] Hestbaek L, Leboeuf-Yde C, Kyvik KO, Manniche C (2006). The course of low back pain from adolescence to adulthood: eight-year follow-up of 9600 twins. Spine.

[5] Ståhl M, Kautiainen H, El-Metwally A, Häkkinen A, Ylinen J, Salminen JJ, Mikkelsson M (2008). Non-specific neck pain in schoolchildren: prognosis and risk factors for occurrence and persistence. A 4-year follow-up study. Pain.

[6] Nelson MC, Neumark-Stzainer D, Hannan PJ, Sirard JR, Story M (2006). Longitudinal and secular trends in physical activity and sedentary behavior during adolescence. Pediatrics.

[7] Hakala PT, Rimpelä AH, Saarni LA, Salminen JJ (2006). Frequent computer-related activities increase the risk of neck-shoulder and low back pain in adolescents. Eur J Public Health.

[8] Karvonen S, Vikat A, Rimpelä M (2005). The role of school context in the increase in young people's health complaints in Finland. J Adolesc.

[9] School Health Promotion Study 2011: Changes in living and school conditions, health and health habits in Finnish schoolchildren from 2000/2001 to 2010/2011. (Kouluterveyskyselyn tuloksia nuorten elinoloista, kouluoloista, terveydestä, terveystottumuksista sekä oppilas- ja opiskelijahuollosta vuosina 2000/2001 - 2010/2011) http://info.stakes.fi/kouluterveyskysely/FI/tulokset/index.htm

[10] Pallesen S, Hetland J, Sivertsen B, Samdal O, Torsheim T, Nordhus IH (2008). Time trends in sleep-onset difficulties among Norwegian adolescents: 1983-2005. Scand J Public Health.

[11] Mollet GA, Harrison DW (2006). Emotion and pain: a functional cerebral systems integration. Neuropsychol Rev.

[12] Petersen S, Brulin C, Bergstrom E (2006). Recurrent pain symptoms in young schoolchildren are often multiple. Pain.

[13] Carnes D, Parsons S, Ashby D, Breen A, Foster NE, Pincus T, Vogel S, Underwood M (2004). Chronic musculoskeletal pain rarely presents in a single body site: results from a UK population study. Rheumatology.

[14] Ståhl MK, El-Metwally AA, Mikkelsson MK, Salminen JJ, Pulkkinen LR, Rose RJ, Kaprio JA (2013). Genetic and environmental influences on non-specific neck pain in early adolescence: A classical twin study. Eur J Pain.

[15] El-Metwally A, Mikkelsson M, Ståhl M, Macfarlane GJ, Jones GT, Pulkkinen L, Rose RJ, Kaprio J (2008). Genetic and environmental influences on non-specific low back pain in children: a twin study. Eur Spine J.

[16] Mikkelsson M, Kaprio J, Salminen JJ, Pulkkinen L, Rose R (2001). Widespread pain among 11-year-old Finnish twin pairs. Arthritis Rheum.

[17] Ståhl M, Mikkelsson M, Kautiainen H, Häkkinen A, Ylinen J, Salminen JJ (2004). Neck pain in adolescence. A 4-year follow-up of pain-free preadolescents. Pain.

[18] Rees CS, Smith AJ, O'Sullivan PB, Kendall GE, Straker LM (2011). Back and neck pain are related to mental health problems in adolescence. BMC Public Health.

[19] Takala EP, Viikari-Juntura E, Moneta GB, Saarenmaa K, Kaivanto K (1992). Seasonal variation in neck and shoulder symptoms. Scan J Work Environ Health.

[20] Landis JR, Koch GG (1977). The measurement of observer agreement for categorical data. Biometrics.

[21] Jeffries LJ, Milanese SF, Grimmer-Somers KA (2007). Epidemiology of adolescent spinal pain: a systematic overview of the research literature. Spine.

[22] Kjaer P, Wedderkopp N, Korsholm L, Leboeuf-Yde C (2011). Prevalence and tracking of back pain from childhood to adolescence. BMC Musculoskelet Disord.

[23] Auvinen JP, Paananen MV, Tammelin TH, Taimela SP, Mutanen PO, Zitting PJ, Karppinen JI (2009). Musculoskeletal pain combinations in adolescents. Spine.

[24] Härmä AM, Kaltiala-Heino R, Rimpelä M, Rantanen P (2002). Are adolescents with frequent pain symptoms more depressed?. Scan J Prim Health Care.

[25] Szpalski M, Gunzburg R, Balague F, Nordin M, Melot C (2002). A 2-year prospective longitudinal study on low back pain in primary school children. Eur Spine J.

[26] Diepenmaat AC, van der Wal MF, de Vet HC, Hirasing RA (2006). Neck/shoulder, low back, and arm pain in relation to computer use, physical activity, stress, and depression among Dutch adolescents. Pediatrics.

[27] Prins Y, Crous L, Louw QA (2008). A systematic review of posture and psychosocial factors as contributors to upper quadrant musculoskeletal pain in children and adolescents. Physiother Theory Pract.

[28] Auvinen JP, Tammelin TH, Taimela SP, Zitting PJ, Jarvelin MR, Taanila AM, Karppinen JI (2010). Is insufficient quantity and quality of sleep a risk factor for neck, shoulder and low back pain? A longitudinal study among adolescents. Eur Spine J.

[CR29] The pre-publication history for this paper can be accessed here:http://www.biomedcentral.com/1471-2474/15/296/prepub

