# Supplementary figures and images for: Time trends in single versus concomitant neck and back pain in finnish adolescents: results from national cross-sectional surveys from 1991 to 2011
Source: BMC Musculoskelet Disord. 2014 Sep 5;15:296. doi: 10.1186/1471-2474-15-296 (PMC4161855; doi:10.1186/1471-2474-15-296)

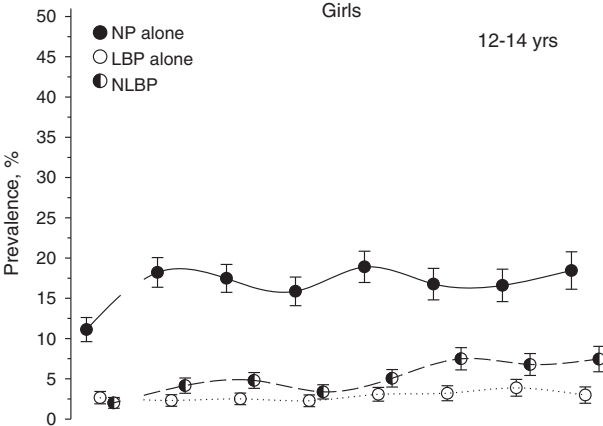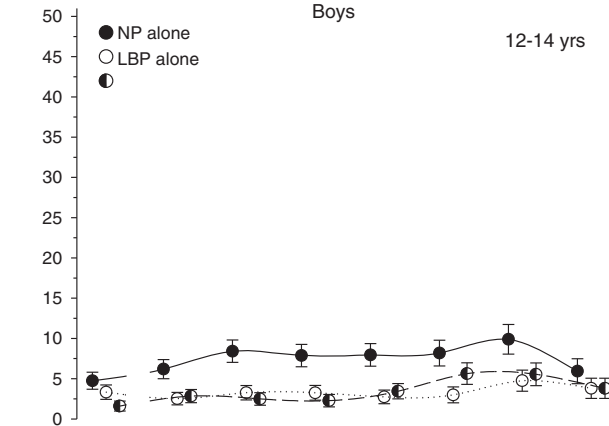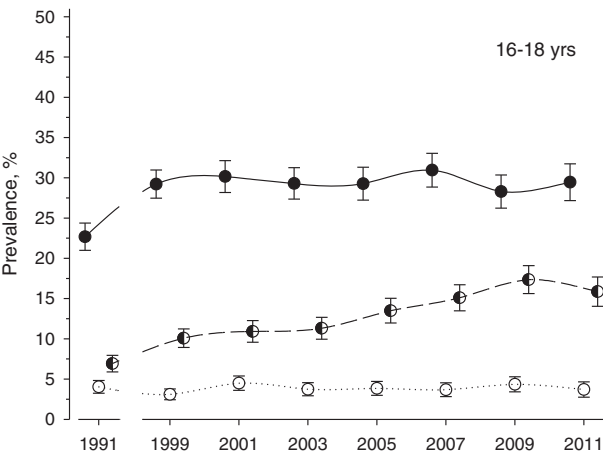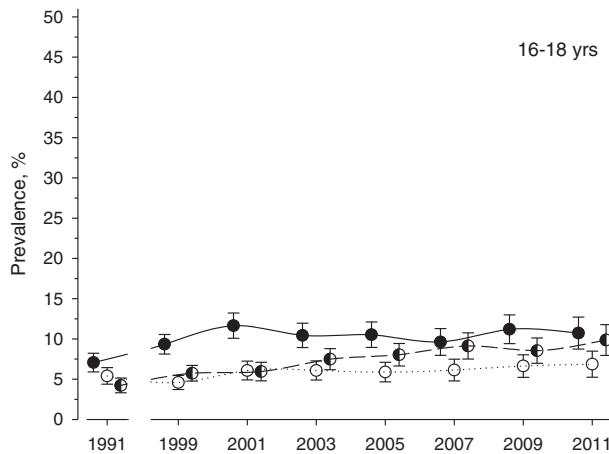

Supplement: Supplementary file 2 — Authors’ original file for figure 1 [file 12891_2014_2238_MOESM2_ESM.pdf]
